# LGBTQ+ Persons’ Experiences of Parenthood in the Context of Maternal and Child Health Care: A Meta-ethnography

**DOI:** 10.1177/23333936231181176

**Published:** 2023-06-20

**Authors:** Charlotte Haugland, Bente Kristin Høgmo, Terese Elisabet Bondas

**Affiliations:** 1 University of Stavanger, Norway

**Keywords:** empowerment, nursing, LGBTQ+ parents, meta-ethnography, parenthood

## Abstract

This study aims to integrate and synthesize knowledge of lesbian, gay, bisexual, transgender and queer (LGBTQ+) persons’ experiences of parenthood in the context of maternal and child health care. For nurses to provide optimal care for LGBTQ+ parents, we need to derive knowledge from their perspectives. An interpretive meta-synthesis approach, meta-ethnography, was chosen for this study. A lines-of-argument synthesis based on four themes was developed: (1) Entering the world of LGBTQ+ parenthood; (2) The emotional journey in LGBTQ+ parenthood; (3) Struggling with the system as a LGBTQ+ parent and (4) A need to expand the knowledge horizon of LGBTQ+ parenthood. The overarching metaphor, “To be recognised as parents, unique and good enough, like everybody else,” reflects how recognition and inclusion may support LGBTQ+ persons in their parenthood and broaden the understanding of parenthood. Knowledge of the LGBTQ+ family needs to be given greater attention in maternity and child health care settings, and in education and health policies.

## Introduction

Parenthood by lesbian, gay, bisexual, transgender and queer persons (LGBTQ+) is gradually becoming more common, due to changes in biotechnological opportunities in reproduction and gender transition, social acceptance of LGBTQ+ persons, and changes in legislation (Carone et al., 2021; Goldberg, 2023). The pathway to parenthood is more cumbersome for LGBTQ+ persons than it is for heterosexual couples (Goldberg, 2023) and one must therefore assume that the children are strongly wished for. Despite changes in society, LGBTQ+ parents still deal with prejudice and discrimination against sexual minorities, as well as a lack of knowledge among healthcare professionals (Hafford-Letchfield et al., 2019; Leal et al., 2021). The organization of maternal and child health care (MCHC) differ from an international perspective, but midwives and public health nurses are vital for the services (World Health Organization, United Nations Population Fund, World Bank & United Nations Children’s Fund (UNICEF), 2015). The nurses support families, with special attention to relationships and parenting. The MCHC may also organize family and childbirth education and support groups.

In this study, the initialism LGBTQ+ is an umbrella term for lesbian, gay, bisexual, transgender and queer. A lesbian is a woman who is romantically and/or sexually attracted to women. A gay man is romantically and/or sexually attracted to men, while a bisexual person is romantically and/or sexually attracted to more than one gender (Stonewall, n.d.). Transgender is an umbrella term for a person whose gender identity and/or gender role do not fit comfortably with the sex that was assigned at birth (Stonewall, n.d.). Queer is a historical term for gay men, lesbian women, or gender non-conforming persons who may not fit traditional ideals of gender, sexuality, or gender identity. This word has more recently become a term of self-identification for people who do not identify with the binary terms traditionally describing sexual orientation. The + sign leaves room for other sexual identities and orientations not covered in the acronym (Stonewall, n.d.). The term LGBTQ+ reflects the definitions used by participants in the studies, but also recognizes that others related to this community may not identify themselves with these definitions.

Knowledge of LGBTQ+ persons’ experiences of parenthood are necessary and may contribute valuable information that can assist nurses and other health care professionals, educators, and political authorities in developing policies, strategies and practices that promote family and community health. LGBTQ+ parents and their families should receive knowledgeable, equal, and non-prejudiced nursing care.

## Background

Parenthood has been described in terms of empowerment as well as challenges, and there is a need to prepare for the changes in life and a need for support in parenthood (Delicate et al., 2018). Like parents in heterosexual relationships, LGBTQ+ parents may experience a decline in well-being and relationship quality in the transition to parenthood (Goldberg et al., 2014). Diversities in family composition and pathways to parenthood, mean that parents often find themselves in a situation where they must explain their family constellation (Gartrell et al., 2019; Haines et al., 2018) and justify the quality of their parenthood (Bos & Gartrell, 2020; Malmquist & Nelson, 2014). Several studies describe lesbian families and their processes of becoming parents or experiences with care (Dahl et al., 2013; Gregg, 2018), revealing the complex nature of lesbian women’s experience of becoming mothers and feeling acknowledged and recognized. Research shows that gay parents more often than lesbian persons challenge the perception related to gender and parenthood, and that gay fathers experience greater challenges and negative assumptions in relation to parenting abilities (Carneiro et al., 2017). In their review, Stotzer et al. (2014) found that transgender parents had a need for available resources or support regarding family planning, childcare and networking with other parents.

An important function of nursing is to assist people in managing life transitions (Meleis et al., 2000), which underpins the importance of nurses having insight into LGBTQ+ parents’ experiences of parenthood, to be able to offer support. Nurses are important public health practitioners as they play a significant role in promoting public health, due to their multi-disciplinary knowledge, competence, and health promotion experience (Kemppainen et al., 2013). Høgmo et al. (2023) describe how public health nurses are in a unique position to provide guidance to parents. However, LGBTQ+ parents still suffer discrimination and stigmatization and may experience lack of support within maternity and child healthcare due to heteronormativity within the systems and lack of competency by health care professionals (Appelgren Engström et al., 2018). Furthermore, Heise et al. (2019) claim that gender inequality and restrictive gender norms reinforced and reproduced by healthcare may have serious implications for health.

Parental empowerment can be seen as a process through which families access knowledge, skills and resources that enable them to gain positive control of their lives (Singh et al., 1995). Empowerment theory refers to a dynamic understanding directed at solutions, growth, and development (Halvorsen et al., 2020), and was chosen as the theoretical perspective for this study. Empowerment is rooted in civil rights and social action and aims to increase the power, autonomy, and influence of exposed groups (Halvorsen et al., 2020). World Health Organization (2021, p. 14) defines empowerment as “a social, cultural, psychological or political process through which individuals and social groups are able to express their needs, present their concerns, devise strategies for involvement in decision-making and achieve political, social and cultural action to meet those needs.” Wallenstein (2006) describes empowerment as a sense of mastery over one’s own life through increased coping mechanisms, skills, and ability to influence one’s life.

There is fragmented knowledge of LGBTQ+ persons’ experiences of parenthood in a maternal and child health care context, and this omission limits our understanding of how persons with diverse sexualities and gender expressions experience this important life event. We have chosen to integrate parents from all previously mentioned LGBTQ+ definitions, as they still suffer discrimination and stigmatization and may experience a lack of support due to heteronormativity within the MCHC system and a lack of competence among nurses and other health care professionals (Appelgren Engström et al., 2018; Röndahl et al., 2009). The term LGBTQ+ reflects the definitions used by participants in the studies, but also recognizes that others related to this community may not identify themselves within these definitions.

## Aim

This meta-ethnography aims to integrate and synthesize qualitative studies that illuminate LGBTQ+ parents’ experiences of parenthood in a maternal and child health care context. The goal is to increase knowledge that may contribute to a better understanding of how to support these families. The research question is: How do LGBTQ+ persons experience their parenthood in a maternal and child health care context?

## Methods

### Design

Meta-ethnography (Noblit & Hare, 1988) was chosen to synthesize the findings from the individual qualitative studies. The method has the potential to interpret and deepen the understanding of the findings in a reflective and systematic way resulting in a conceptual understanding of a phenomenon (France, Cunningham et al., 2019; Noblit & Hare, 1988). The study follows Noblit and Hare’s (1988) method, consisting of seven overlapping phases, as a nonlinear interpretive approach. The phases are: Getting started (1), deciding what is relevant (2), reading the studies (3), determining how the studies are related (4), translating the studies into one another (5), synthesizing translations (6), and finally, expressing the synthesis (7),

In addition, the eMERGe reporting guidance developed by France, Cunningham et al. (2019) was used to improve the clarity and integrity of meta-ethnographic reporting.

### Data Collection and Analysis

#### Phase 1—Getting Started

A curiosity for the topic arose when one of the authors, (CH), became aunt to two girls, who have two mothers. We all shared an interest to deepen the knowledge of supporting parenthood for families, as practitioners and educators. Nurses will encounter a variety of family constellations to care for. However, during public health nurse training over different decades in various countries, none of the three authors had experienced any introduction to the LGBTQ+ community or family constellations.

#### Phase 2—Deciding What is Relevant

We developed the inclusion and exclusion criteria to allow for a specific, systematic, and thorough search. As the search process revealed relevant studies, it was easier to see if the inclusion and exclusion criteria needed refinement (see Table 1).

**Table 1.** Inclusion and Exclusion Criteria.

Inclusion criteria: • Peer-reviewed original qualitative studies • Parents who identify themselves within the LGBTQ+ definition (Lesbian, gay, bisexual, transgender, queer) and their perspectives or experiences of parenthood in the context of maternal and child health care • English or Scandinavian Languages Exclusion criteria: • Quantitative studies • Mixed-methods studies • Studies from the perspectives of heterosexual parents • Studies from the perspectives of healthcare professionals or healthcare students • Studies of the decision-making process and choices prior parenthood • Studies on LGBTQ+ parents and parenthood in combination with an added topic like reproduction, birth experience, adoption, religion, ethnicity, politics, school-age children or older.

A search strategy was developed together with a university librarian. The following terms were applied: LGBTQ+ parents OR lesbian/gay/bisexual/transgender/queer parents AND maternity and care services OR child health service AND experience AND parenthood. In addition, MESH terms and CINAHL headings were used to maximize the success of the search strategy. Three databases of peer-reviewed literature were considered relevant to the research question: Academic Search Premier, CINAHL and Scopus with a cut-off in November 2021. In addition, a complementary search was performed in Google Scholar.

**Figure 1.**
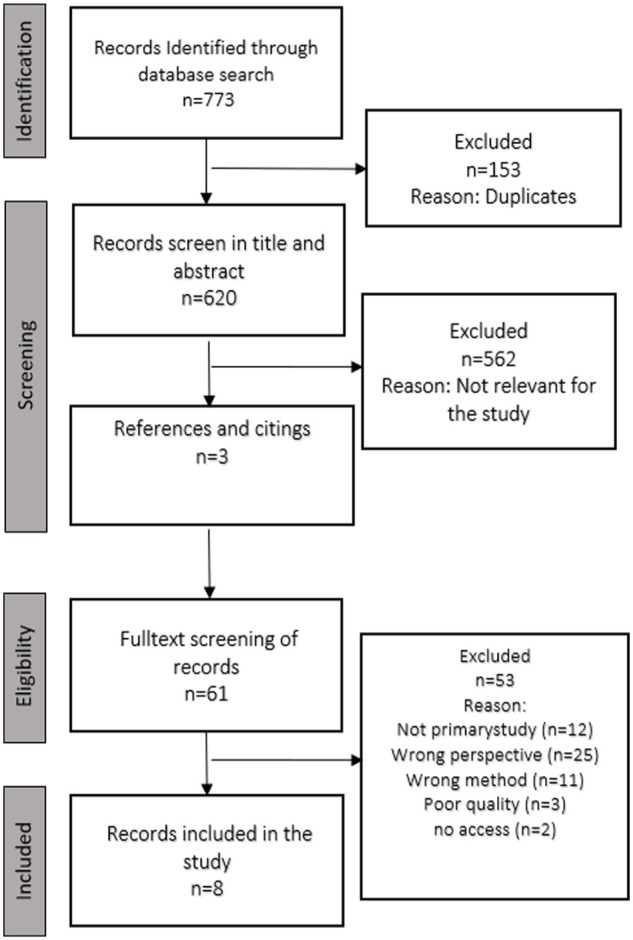
PRISMA flow chart.

The selection of primary studies was conducted in stages to ensure a systematic and transparent process and was discussed with all three authors for clarification and agreement. The PRISMA flowchart (Page et al., 2021) was used to record the process of the inclusion and exclusion of studies (see Figure 1). A total of 773 records were identified through database searches. After the removal of duplicates (using Zotero), 620 studies were included in the screening process. First, 562 studies were excluded based on title and abstract. Citations and references were searched, which provided three additional articles. Thereafter, the remaining 61 studies were read in full text and re-examined in relation to the criteria. At this stage, 53 articles were excluded. Lastly, the remaining eight articles underwent qualitative assessment on aspects such as aims, methods, research ethics using the Critical Appraisal Skills Program (CASP), a checklist for qualitative research (2018). All studies met eight of the nine quality criteria (see Supplemental File for CASP table). Only one study (Dahl & Malterud, 2015) met criteria #6: Has the relationship between researcher and participants been adequately considered? There was insufficient information to make a determination on this criterion in the remaining studies. Based on the quality review, it was decided to include all eight articles.

#### Phase 3—Reading the Studies

In this phase, CH repeatedly read the findings line-by-line, presented in the studies included, taking notes of findings and citations described or developed by authors of primary studies (France, Cunningham et al., 2019). A presentation of the characteristics of the studies includes author, year, country, aim, design, participants, and main findings (see Table 2). Two of the studies were derived from the same primary data (Kerppola et al., 2019, 2020).

**Table 2.** Presentation of the Included Studies.

Author/year	Country	Aim	Design	Participant characteristics	Main findings
Kerppola et al., 2020 INDEX STUDY	Finland	To describe the supporting factors for LGBTQ parents’ empowerment in maternity and child healthcare from the perspective of self-identified LGBTQ parents in Finland.	A qualitative inductive design - inductive content analysis	22 Parents; Thirteen lesbian, four gay, one identified as transgender, - one as bisexual, - two as nonbinary. - Both, single- and multiple-time parents were included, and eleven were nonbiological parents - Two were not guardians of their children - Demographics, such as age, work, or education, were not requested. - Lived in several different areas in Finland - From Nordic countries and 2 had immigrant background	LGBTQ parents are committed to start their own families and embrace parenthood. This meant facing discrimination attitudes, having feelings of not being good enough and required good self-knowledge and strong self-esteem. Being supported are related to human rights (dignity), healthcare policy and structures which involves recognition and acknowledgment included gender identity and parenting role, and that professionals demonstrate understanding and respect which contributed to establish trusting and open relationships. Lack of knowledge was conveyed via heteronormativity in communication, forms, and group meetings. Parenthood can be challenging, and parents stated that they needed to be taken seriously, given practical support and valuable feedback in an inclusive and sensitive environment with good coordination such as continuity of care and good accessibility.
Andersen et al. (2017)	Sweden	To describe lesbian, gay, and bisexual parents’ experiences of nurses’ attitudes in child healthcare	A qualitative inductive design, semi structured interviews, qualitative content analysis	Fourteen informants: Eleven mothers, three fathers - One family included two mothers and one father - Thirteen same-sex families. - Three informants were not born in Sweden	LGB parents experienced marginalization which originated from a lack of knowledge about LGB families, family constellations and roles within the family. Written material was perceived as heteronormative. The nurses were considered nonchalant and disrespectful and the nonbiological parents were not viewed as to be as important. Negative experiences of being forced to “come out.” However respectful and inclusive experiences were related to the parents being treated like a unique individual and that the nurses demonstrated sensitivity, professionalism, by for example using correct vocabulary, and acceptance when they were open-minded, showed genuine interest, saw them as a family, and included all parents. This made the parents feel validated and accepted in their parental role.
Appelgren Engström et al. (2019)	Sweden	To get a deeper understand of how mothers in same-sex relationships think and reason about their parenthood in terms of gender equality and how they experience early parental support from child healthcare professionals.	Grounded theory, Follows guidelines for qualitative research (COREQ), Semi-structured interviews	Twenty women	With support and information from the healthcare professionals, the co-mother could have been encouraged to breastfeed the baby. Breastfeeding contributed to natural attachment with the baby. Attachment was also developed by spending time with and being physically close to the child. There is a difference in attachment being birthmother or co-mother. The families sometime felt they had to justify their family structure. Important that the co-mother felt acknowledged, spoken to, and seen as an equal parent and received the same support as the birthmother. The term “Partner” did not validate the parental role. A need for a network with other same-sex families.
Chapman et al. (2012)	Australia	To explore and describe the experiences of LGT parents accessing health care for their children	Descriptive qualitative study, semi-structured interviews,	Eleven informants: Seven lesbian couples, one gay person couple, three couples for which one was transgender (one MTF, two FTM)	LGT parents pointed out that they had to educate healthcare professionals about LGT families which to some was seen as a responsibility but for others felt tiring. Little awareness of respectfully interaction with transgender parents for example using correct pronouns. Previous experiences can make you defensive (to the system). Positive interactions with healthcare were combined with attitude which included professionals not to be judgmental and making assumptions, and focusing on the reason for seeking healthcare, rather than the family constellation. Besides, acknowledged both parents for having equal saying and responsibility for the health care of their child. Despite legal changes the bureaucratic attitudes and positions can be read the rhetoric of forms (heteronormative and exclusive).
Cherguit et al. (2013)	United Kingdom	To explore co-mothers’ experiences of maternity healthcare services in the UK from their partner trying to conceive to post birth care.	Qualitative design, interpretative phenomenological analysis methodology, semi-structured interviews.	Ten co-mothers	Co-mothers felt excluded by the system and struggled to develop a parental identity not being a mother nor a father, having anxiety about becoming a parent and worries about bonding with the baby. The co-mothers anticipated prejudice and discrimination from the staff and interpreted positive experiences as exceptions. It is difficult to know if poor treatment was due to homophobia or incompetence. Heterocentricity was expressed through language, forms, and the system itself which made co-mothers feel excluded and invisible. The co-mothers had positive experiences with MHC staff as the co-mothers felt they treated them equally, felt acknowledged and recognized by the way the staff interacted with them. The co-mothers felt as pioneers and as their own confidence got stronger, it led to more acceptance by the MHC staff.
Dahl and Malterud (2015)	Norway	To explore lesbian co-mothers’ maternity care experiences and implications for the caring encounter	A qualitative interview study	Eleven women - Six participants had given birth before becoming a co-mother - Three had already co-mothered two children	The encounters with healthcare providers were positive and uncomplicated as they seemed aware of the lesbian relationship. Ordinary tokens of recognition created feelings of being included. Lesbian self-confidence played a key role in awkward encounters. Being neither father nor biological mother sometimes challenged parental identity. Being women helped co-mothers understand what their partner went through, even though they had to find new ways of mothering than if they had given birth themselves. Co-mothers addressed themselves with different terms and some concepts was perceived as unnatural or excluding. Terms like “co-mother” was perceived as a bureaucratic concept. The parental identity was defined by their relationship with the child.
Kerppola et al. (2019)	Finland	To describe parental empowerment in maternity and child healthcare in Finland from the perspective of self-identified LGBTQ parents	A qualitative inductive design - inductive content analysis	Twenty-two parents; - One identified as transgender, - one as bisexual, - two as nonbinary. - Both, single- and multiple-time parents were included, and eleven were nonbiological parents - Two were not guardians of their children - Demographics, such as age, work, or education, were not requested.	Parental empowerment is tied to recognition and acknowledgment, regarding the ability to define themselves as an LGBTQ family and how to be “a parent”. This includes attention to language, being noticed and considered as well as having feelings of “not fitting in.” Cooperation and interaction between professionals and the parents included gaining knowledge, support and help along with professionals’ capacity to recognize parents needs and own expertise which influence parents’ involvement and willingness to participate in appointments. Equality is having a sense of security, excellent quality of care on professionals’ competence and skills with respect for the entire family, good access to healthcare and experience parity and be treated like a normal family.
McKelvey (2014)	USA	To explore postpartum experiences of nonbirth lesbian mothers within their first year of motherhood.	A metastory, a narrative analysis centers on the story as the object of investigation, Riessmann’s method of thematic narrative analysis.	Ten participants	Both positive and negative experiences with interacting with the healthcare professionals from being included, acknowledge, and respected to be ignored, not recognized, and had to advocate for their partner. Nursing is the major difference between mothers - using a bottle can help the co-mother feel more involved. A name validated them as a parent. They felt unprepared for motherhood despite anticipating motherhood for many years. Becoming a mother taught participants a great deal about themselves. Having fears about not bonding with the baby, not being recognized as a mother, fear of judgmental attitude if she “breastfeeds” the baby.

#### Phase 4—Determining How the Studies Are Related

After repeated reading of the studies and discussion with all three authors, the findings were juxtaposed to determine how they were related. According to Noblit and Hare (1988), the relationship between the studies appears as either reciprocal (similar), refutational (in opposition) or in line of argument (cumulative). After the preliminary analysis, the findings were found to be reciprocal.

#### Phase 5—Translating the Studies Into One Another

The translation process is a vital part of conducting a meta-ethnography and unique compared to other qualitative synthesis methodologies (France, Uny et al., 2019). Translation is idiomatic rather than literal (Noblit & Hare, 1988). It refers to the process of translating the finding or citation from one study and recognizing them in another, even though they are not expressed using the same words but represent a meaning. The aim is to arrive at concepts or metaphors which embrace more than one study (France, Uny et al., 2019). We created a matrix to present an overview and maintain a systematic base for the analysis. In Table 3, the translation process is exemplified whereby an index study characterized as rich (Kerppola et al., 2020) was placed in the first column as a starting point (France, Uny et al., 2019). The findings were sorted and grouped together with similar findings from other studies. This was a translation process, going back and forth between the findings, the primary studies, and the emerging synthesis, which enabled the findings to be coded and subsequently sorted toward themes and an integrated synthesis.

**Table 3.** Examples of the Translation Process.

Kerppola et al. (2020) (INDEX STUDY)	Andersen et al. (2017)	Appelgren Engström et al. (2019)	Chapman et al. (2012)	Cherguit et al. (2013)	Dahl and Malterud (2015)	Kerppola et al. (2019)	McKelvey (2014)	Authors’ translation
stand up and explain themselves	be forced to “come out”	-	“But why should you have to explain yourself to everybody”	-	had to explain if they were the baby’s biological mother	-	To be recognized as a mother, they must come out as a lesbian. Nonbirth mothers had to frequently explain their family to strangers	Forced to “come out” and stand up for themselves
“She was always willing to hear how we are, are we okay, and was there anything we need, like help or something, and I felt she was interested about our well-being.”	treated them with sensitivity, professionalism, and acceptance “The CHC was kind of a waterhole because it was there, we were. . .	-	health professionals did not judge or make assumptions about them	positive interactions had an eroding effect on their expectations of prejudice and discrimination	most of their encounters with healthcare providers had been positive and uncomplicated	easy to talk to, nonjudgmental and fair.	praised HCPs for demonstrating genuine empathy and happiness for their new families	Trusting relationship were developed due to positive interpersonal values/skills and professionalism

Kerppola et al., (2020): Index study, thereafter alphabetical order.

#### Phase 6—Synthesizing Translations

After the translation process was completed the themes and metaphors that had been extracted from the findings were analyzed. A meta-ethnography is interpretative, with the potential to create and offer metaphors of deeper and broader understanding (Bondas et al., 2017). Through each study, a new layer of understanding was added. In the interpretative process all the authors reflected upon their pre-understanding, making it important to maintain a critical approach, which contributed to discovering new understandings and different perspectives. Four themes were developed. Guided by Noblit and Hare (1988), and the guidelines by France, Cunningham et al. (2019), the translation proceeded from reciprocal translation to lines-of-argument synthesis, as the findings were tied to one another. Refutations were not found, as there were no contradictory patterns between findings. Table 4 shows the lines-of arguments synthesis.

**Table 4.** From Translations to Themes and Synthesized Metaphor.

Translation	Subthemes	Themes	Synthesized metaphor
Forced to “come out and stand up for themselves”	The right to a LGBTQ+ identity	Entering the world of LGBTQ+ parenthood	To be recognized as parents, unique and good enough, like everybody else
Having a LGBT+ identity influenced the encounters			
Protecting oneself against negative experiences			
“In the eye of the beholder”			
A need for a supportive environment	The importance of belonging to a supporting community		
Support and connection to a LGBT+ community was important			
Working together			
Understanding the limitations and benefits of relationships to family and friends			
Being accepted and treated like everybody else	To be like everyone else, as parents and family		
Open communication			
Being a “normal” family			
Becoming a parent require self-knowledge and strong self-esteem	Becoming a parent	The emotional journey in parenthood	
Parental empowerment grow in positive interactions with healthcare professionals			
Informed decision-making creates a feeling of autonomy			
Developing parental identity			
Inclusion Not an easy pathway to parenthood Parenthood embraces conflicting emotions Parenthood can provide personal growth			
Developing a bond with the baby	Developing attachment to the child		
The bonding process is different			
Equality in parenthood	Changes in the couple’s relationship		
Committed to making it work			
Forbidden thoughts			
Relationship in transition			
Non-birth mothers’ ability to try breastfeeding if informed and supported	A need for more knowledge and greater awareness	A need to expand the knowledge horizon of LGBTQ+ parenthood	
Need of well-educated professionals for support			
A need for knowledge and awareness about LGBT+ parents’ specific needs and services			
Individualized information was helpful			
Access to services			
A need to redefine the family concept	A need for greater understanding of various family constellations		
Lack of understanding family constellations			
Openness leads to acceptance			
Recognition and acknowledgment in the encounters with healthcare professionals	A need for a personal and trusting relationship with healthcare professionals		
Trusting relationships were developing due to positive interpersonal values, skills, and professionalism			
A need to trust one another’s expertise			
Small gestures matter			
A need for openness toward gender variety	The feeling of not fitting in	Struggling with the system as a LGBTQ+ parent	
Struggling to find a parental identity as a non-biological parent			
Felt marginalized as the system is too heteronormative			
Prejudice and heterosexism were displayed in negative attitudes			
The feeling of not fitting in			
Fear of the system	The right to be a family – fear and struggle		
Advocating for the right to be a family			
Struggle with a non-heteronormative identity			
Structural power within the system			
An inclusive language confirmed the parents in their parenthood	The meaning of responsive language		
The power of language			

## Results

An overarching metaphor “To be recognised as parents, unique and good enough, like everybody else” was constructed during the interpretive analysis, based on the four themes and sub-themes: (1) Entering the world of LGBTQ+ parenthood; (2) The emotional journey in LGBTQ+ parenthood; (3) Struggling with the system as a LGBTQ+ parent, and (4) A need to expand the knowledge horizon of LGBTQ+ parenthood.

### Entering the World of LGBTQ+ Parenthood

The first theme “Entering the world of LGBTQ+ parenthood” consists of three sub-themes: (a) To be like everyone else as parents and family; (b) The right to an LGBTQ+ identity; and (c) The importance of belonging to a supportive community.

#### To be Like Everyone Else as Parents and Family

LGBTQ+ parents wanted to be a family (Kerppola et al., 2019). They wished to be treated and accepted like everybody else (Andersen et al., 2017; Dahl & Malterud, 2015; Kerppola et al., 2019, 2020; McKelvey, 2014) and be part of the important moments of their child’s life (Cherguit et al., 2013). They felt validated and respected when all parents were included and received the same support as other parents (Andersen et al., 2017; Kerppola et al., 2019). This substantiated the feeling of being respected for who they were and that their family was seen as exactly that – a family (Andersen et al., 2017; McKelvey, 2014). This included being able to communicate with respect and trust to share concerns with each other and with MCHC professionals (Kerppola et al., 2020).

#### The Right to a LGBTQ+ Identity

LGBTQ+ parents described how they had to “come out” as LGBTQ+ parents and explain to strangers their family constellation, and thereby had to stand up for themselves (Andersen et al., 2017; Dahl & Malterud, 2015; Kerppola et al., 2020; McKelvey, 2014). One parent questioned “[. . .] why should you have to explain yourself to everybody” (Chapman et al., 2012, p. 1132). The individual’s self-confidence and life experience could be seen as a strength and helped them to live as LGBTQ+ families (Kerppola et al., 2020) and shape their lives, which gave a sense of security (Dahl & Malterud, 2015).

Previous negative experience of discrimination had an impact on how LGBTQ+ parents encountered MCHCs. For some parents, it meant encountering MCHCs with low expectations, to protect themselves (Chapman et al., 2012; Cherguit et al., 2013). The parents’ low expectations could be so strong that when they experienced a positive encounter, this was seen as an exception (Cherguit et al., 2013). Furthermore, LGBTQ+ parents experienced how they were often in a position where they had to take an educational role that could be perceived as tiring (Chapman et al., 2012; Kerppola et al., 2019). Some described themselves as pioneers with a responsibility to pave the way for future LGBTQ+ parents (Chapman et al., 2012; Cherguit et al., 2013).

#### The Importance of Belonging to a Supportive Community

A sense of belonging was essential for the LGBTQ+ persons’ experience of parenthood. A supporting environment meant a place where they could feel confident, relaxed, and comfortable, and could work together for the benefit of the family’s well-being (Kerppola et al., 2020). They saw it as the nurses’ duty to provide a safe environment (Kerppola et al., 2019), which also included a sense of security, voluntary disclosure, and dignity (Kerppola et al., 2020). They felt supported when nurses were open-minded, had a positive attitude, focused on the strength within the family, and explicitly encouraged them and told them they were good parents (Kerppola et al., 2020).

Furthermore, the parents expressed a need for a connection to an LGBTQ+ community where they could discuss and exchange experiences, and their child could see that there were other families like theirs (Appelgren Engström et al., 2019). The importance of belonging also required an understanding of the benefits and limitations that family and friends could have for the LGBTQ+ family’s experiences of a supporting community. For some, the family of origin was a resource, but this was not the case for everyone. Therefore, friends were sometimes seen as important “chosen family” members (Kerppola et al., 2020).

### The Emotional Journey in LGBTQ+ Parenthood

The theme “the emotional journey in parenthood” reflects the strong emotions when developing a parental identity. The theme comprises four subthemes: a) Becoming a parent; (b) Developing attachment to the child; and (c) Changes in the couple’s relationship.

#### Becoming a Parent

LGBTQ+ parents felt that the decision to become parents was not entirely up to them, as “the choice of having a family lies in the system” (Cherguit et al., 2013, p. 1271). Although the desire was strong and longed for (Kerppola et al., 2020), they felt unprepared and overwhelmed as parents (McKelvey, 2014). They experienced parenthood as rewarding and thrilling on the one hand, but also associated it with feeling isolated and lonely, and exhausted and terrified, on the other hand (Kerppola et al., 2020; McKelvey, 2014). However, parenthood was also perceived as an opportunity for personal growth and self-discovery (McKelvey, 2014). To become a parent, it was necessary to have self-knowledge and strong self-esteem (Kerppola et al., 2020). The support and encouragement which the LGBTQ+ parents received from the nurses had a significant impact on their ability to develop a sense of parental empowerment. This was associated with the capacity of nurses to see the needs and wishes of LGBTQ+ parents (Kerppola et al., 2019, 2020; McKelvey, 2014). The LGBTQ+ parents’ sense of autonomy grew when they felt included in decision-making, were well-informed, and were able to influence the service situation by choosing topics important to them in the consultation (Kerppola et al., 2019, 2020). Encouragement to ask questions was viewed as positive (Kerppola et al., 2019).

Findings indicate that the experience of inclusion in the way professionals approached them gave the LGBTQ+ parents a feeling of being recognized as parents and a sense of belonging (Appelgren Engström et al., 2019; Dahl & Malterud, 2015; Kerppola et al., 2019; McKelvey, 2014). One lesbian co-mother recalled a question about a rash from a nurse: “His skin is a little dry but do any of you have a problem with eczema, she asked. I thought she was so sweet, so I just answered the question” (Dahl & Malterud, 2015, p. 171). The heteronormative vision of parenthood did not always resonate with the LGBTQ+ parents’ experience of parenthood: “The maternal role did not resonate with her; however, she absolutely felt like a parent” (McKelvey, 2014, p. 108).

#### Developing Attachment to the Child

Developing a bond to the child was described as an emotional experience. LGBTQ+ parents worried about the bonding and whether there would be a difference between the parents (Cherguit et al., 2013; Kerppola et al., 2020; McKelvey, 2014). Developing a parental identity was defined by the relationship with the child and not by biological ties (Dahl & Malterud, 2015; McKelvey, 2014). Mothers who had experienced this from both sides said that it took longer to develop a bond with a child to whom they had not given birth (Appelgren Engström et al., 2019) and that they had to find a new way to be a mother for their child (Dahl & Malterud, 2015). Breastfeeding contributed to a natural attachment but was not crucial for the bonding process (Appelgren Engström et al., 2019; Dahl & Malterud, 2015). However, one co-mother described how non-nutritive nursing eased her insecurities and helped her to bond more easily with the baby (McKelvey, 2014), while for other LGBTQ+ parents it was important that both could feed the baby (Appelgren Engström et al., 2019; McKelvey, 2014). LGBTQ+ parents experienced that by spending time with and being physically close to the child, such as skin-to-skin contact, they developed an attachment (Appelgren Engström et al., 2019; McKelvey, 2014).

#### Changes in the Couple’s Relationship

Becoming parents imposed tremendous strain on a couple’s relationship (McKelvey, 2014). Some experienced grief about the changes in the relationship (Kerppola et al., 2020), as the partner was less available (McKelvey, 2014). One parent stated that she experienced jealousy toward her partner, due to the bond that breastfeeding gave (McKelvey, 2014), while another parent expressed having what she called forbidden thoughts and shame in wanting to be a birth mother one day (Appelgren Engström et al., 2019). However, the LGBTQ+ parents were committed to making it work for the sake of the child (Kerppola et al., 2020) and to finding ways to support and nurture the family (Kerppola et al., 2020; McKelvey, 2014).

### Struggling With the System as an LGBTQ+ Parent

The findings indicate how LGBTQ+ persons felt they had to struggle with the system to be recognized as parents. This theme is based on three sub-themes: (a) The feeling of not fitting-in; (b) The right to be a family*—*fear and struggle; and (c) The meaning of responsive language.

#### The Feeling of Not Fitting-In

The findings indicate that LGBTQ+ parents experienced not fitting-in, since nurses expressed prejudice and heterosexism, displayed as negative attitudes (Cherguit et al., 2013; Kerppola et al., 2020). This was perceived as disrespectful and offensive (Andersen et al., 2017; McKelvey, 2014). The feeling of not fitting-in is substantiated by the information distributed by MCHCs, which is based on heterosexual family constellations (Kerppola et al., 2019). The language used in forms and literature underlined that their family was different from other families (Cherguit et al., 2013; Kerppola et al., 2019, 2020). Furthermore, waiting rooms were often furnished with only two chairs for the parents, which clearly illustrated that families with non-traditional constellations did not fit in (Kerppola et al., 2019). In addition, LGBTQ+ parents experienced the understanding of gender as too narrow and wanted greater openness toward gender variation and the opportunity to define their own gender (Andersen et al., 2017; Appelgren Engström et al., 2019; Kerppola et al., 2019, 2020). The feeling of not fitting-in also had an impact on the parental identity of non-biological parents, as this parental role was more vulnerable and they were exposed to more negative experiences (Andersen et al., 2017; Cherguit et al., 2013; Kerppola et al., 2020). These parents felt misunderstood and ignored (Kerppola et al., 2020) and were afraid of being perceived as not being competent or equal parents (Andersen et al., 2017; Kerppola et al., 2019; McKelvey, 2014).

#### The Right to be a Family—Fear and Struggle

The LGBTQ+ parents described how they approached MCHCs with a great fear of not being recognized as parents, being met with a judgmental attitude regarding their sexuality and family situation, and that there would be no confidence in their authority to make decisions for the child (Cherguit et al., 2013; Kerppola et al., 2019, 2020; McKelvey, 2014). Equality within parenthood meant being acknowledged, included, and spoken to as equal parents (Appelgren Engström et al., 2019; Chapman et al., 2012; Kerppola et al., 2020; McKelvey, 2014). To clarify the family constellation, having the same surname was a way to protect the family (McKelvey, 2014). LGBTQ+ parents experienced how the system influenced their experience of the right to be a family. This is apparent from such terminology as “co-mother,” which seemed to make everything fit the system, but was perceived as unnatural and bureaucratic by the parents (Dahl & Malterud, 2015). In addition, concepts such as “non-birth mother” substantiated a feeling of exclusion in parenthood, as the parental role is defined based on what one is not, rather than what one is (McKelvey, 2014).

#### The Meaning of Responsive Language

Findings in all the included studies showed the importance of language for LGBTQ+ persons’ experiences of being recognized in their parenthood. The LGBTQ+ parents described that nurses did not always use the right words and expressions, but this was not experienced as problematic when the impression was that they tried their best (Andersen et al., 2017; Dahl & Malterud, 2015). In addition, it was important that nurses were responsive to the language used by the families or asked what words the parents themselves wanted to use in their parenting (Andersen et al., 2017; Chapman et al., 2012; Dahl & Malterud, 2015; Kerppola et al., 2019; McKelvey, 2014). The LGBTQ+ parents wanted more openness, allowing them to choose and use the words that made them feel like parents: “She chose to be called ‘mama’ since this is the name that authentically symbolises motherhood to her” (McKelvey, 2014, p. 110).

It appeared from several of the studies that through their language, nurses showed a lack of understanding of the family constellation, since it was often asked “which of you is the parent of the child” or “which of you is the mother,” which led to the non-biological parent feeling ignored and excluded (Appelgren Engström et al., 2019; Chapman et al., 2012; Dahl & Malterud, 2015; Kerppola et al., 2019), and some even felt that they were not seen as parents at all (Kerppola et al., 2019).

### A Need to Expand the Knowledge Horizon of LGBTQ+ Parenthood

A need to expand the knowledge horizon of LGBTQ+ parenthood expresses how LGBTQ+ parents experienced a lack of knowledge among nurses of their family constellation and how that affected their ability to embrace parenthood. This is described in three sub-themes: (a) A need for more knowledge and greater awareness; (b) A need for greater understanding of various family constellations, and (c) A need for a personal and trusting relationship with nurses.

#### A Need for More Knowledge and Greater Awareness

Like other parents, LGBTQ+ parents needed support and guidance on the upbringing of their children regarding routines, caretaking and development knowledge through individualized information, practical support, and advice (Kerppola et al., 2019, 2020). They experienced that the nurses were well-educated within their field (Appelgren Engström et al., 2019; Dahl & Malterud, 2015), but lacked knowledge and experience concerning LGBTQ+ families (Chapman et al., 2012; Dahl & Malterud, 2015). One parent stated, “I think there is an issue of medical professionals needing proper training about a range of issues related to LGBT people and that’s about lesbian sexual health, about different kinds of families, about diversity” (Cherguit et al., 2013, p. 1273). Knowledge concerning transgender people was deficient (Chapman et al., 2012). This meant that the LGBTQ+ parents had to obtain specific information from peers and the internet instead (Kerppola et al., 2020). There was no expectation of detailed knowledge from the nurses, but sufficient knowledge to be able to take care of and support the entire family (Andersen et al., 2017; Appelgren Engström et al., 2019; Kerppola et al., 2019). Non-birth mothers had the ability to breastfeed if informed and supported by nurses (Appelgren Engström et al., 2019; Dahl & Malterud, 2015). However, many were unaware of this possibility or feared judgmental attitudes (Appelgren Engström et al., 2019; McKelvey, 2014).

#### A Need for Greater Understanding of Various Family Constellations

LGBTQ+ parents experienced how nurses lacked an understanding of various family constellations, as the parents experienced a lack of acknowledgment as a family (Andersen et al., 2017; Chapman et al., 2012; Kerppola et al., 2020) and had to justify their family structure (Appelgren Engström et al., 2019). Fear of discriminative attitudes made them reluctant to participate in parental groups (Kerppola et al., 2019). However, despite their concern, LGBTQ+ parents experienced that openness led to acceptance (Cherguit et al., 2013). Being open about the family constellation also reflected how they wanted their children to view the family. There is a need to redefine the family concept and broaden the understanding of the distinct roles within the family (Andersen et al., 2017; Dahl & Malterud, 2015; Kerppola et al., 2019; McKelvey, 2014).

#### A Need for a Personal and Trusting Relationship With MCHC Nurses

The LGBTQ+ parents found that the relationship with nurses was of immense importance to their experience of parenthood (Andersen et al., 2017; Chapman et al., 2012; Kerppola et al., 2019, 2020). A trusting relationship could be developed based on positive interpersonal values and skills, as well as the nurses’ professionalism and demonstration of genuine empathy and happiness for the families (Andersen et al., 2017; Chapman et al., 2012; Cherguit et al., 2013; Dahl & Malterud, 2015; Kerppola et al., 2019, 2020; McKelvey, 2014). The quality of this trusting relationship grew when LGBTQ+ parents experienced nurses who were easy to talk to and who treated them with sensitivity and showed a genuine interest by listening. The parents appreciated consistency and sufficient time to become familiar (Andersen et al., 2017; Chapman et al., 2012; Cherguit et al., 2013; Dahl & Malterud, 2015; Kerppola et al., 2019, 2020; McKelvey, 2014). Furthermore, the experience of trust was enhanced when the parents and professionals managed to trust each other’s expertise (Kerppola et al. 2019, 2020). Correcting brochures or a crib card were experienced as small gestures with a profound impact on being recognized as parents (Andersen et al., 2017; Dahl & Malterud, 2015; McKelvey, 2014).

## The Synthesis

**Figure 2.**
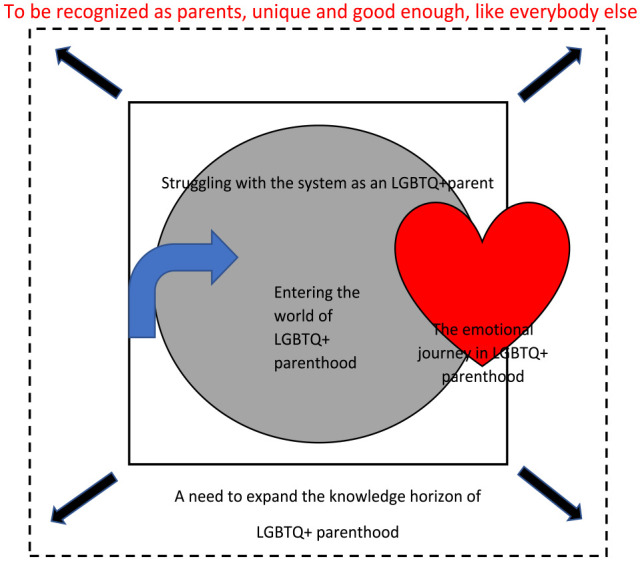
The lines-of-arguments synthesis.

Synthesizing the studies and reflecting on the themes generated a lines-of-argument synthesis (Noblit & Hare, 1988). The synthesis illuminates LGBTQ+ persons’ need to be recognized as parents within their parenthood, which is expressed in this study by the metaphor “To be recognised as parents, unique and good enough, like everybody else” (see Figure 2). *The arrow* represents the entering of LGBTQ+ parenthood. To properly understand their world, as a nurse one needs to enter it and see things from their perspective. *The heart* illustrates the emotional journey of becoming a parent. The parents need to retain their hard-fought-for LGBTQ+ identity and hope to be accepted as parents in a heteronormative world, which can be overwhelming and challenging. *The innermost closed box* represents the struggle to fit into the existing heteronormative society continuing at the MCHCs, which feels limiting and often discriminatory and exclusionary. LGBTQ+ parents experience that they need to justify their parenthood and family constellations. *The arrows and dotted lines* show the need to expand the knowledge horizon to ensure the inclusion of LGBTQ+ parents and different family constellations. This expansion is also required to ensure that nurses can provide quality care and the necessary support for these families, while contributing to the development of the experience of parental empowerment for LGBTQ+ parents.

However, on using the words “like everybody else” in the metaphor there is a risk of not recognizing the distinctive needs of LGBTQ+ parents. This is thus not to be understood as claiming no difference between LGBTQ+ parenting and heterosexual parenting, as if they were just to be looked on as two groups, but rather as acknowledging the specific needs and challenges one can find in any family. Being recognized as parents was experienced as an opportunity for personal growth, where they must find a way to integrate the LGBTQ+ identity with society’s perception of parenthood. An important premise is the need to be recognized and confirmed in their parenthood by being treated and accepted like every other parent. This strengthened the feeling of being supported and belonging to the community as parents and LGBTQ+. “Unique and good enough like everybody else” suggests that LGBTQ+ parents expand the understanding of parenthood, bringing new dimensions to what parenthood can look like and how parenting is defined by the parents’ love and care for the child, and not the biological bonds or the label the system gives us. The struggle which began as a lack of recognition and knowledge generated a distance between the parents and the nurses, due to interpersonal factors such as attitude or disrespectful statements, or systemic factors such as the language used in material, underlining a heteronormative understanding of what family is.

## Discussion

The theoretical perspective of empowerment, based on the three core principles of recognition, participation, and power (Halvorsen et al., 2020; World Health Organization, 2009), provided a framework from which the findings can be examined to deepen the understanding of the synthesis. This meta-ethnography illuminates how LGBTQ+ parents want to be recognized and acknowledged as a family and accepted and respected in their parenthood.

### Recognition of LGBTQ+ Parenthood

Recognition is, according to Honneth’s (1995) theory, a pervasive need that all people have and is essential for identity formation. It is expressed in three forms, as emotional, legal, and social recognition. To be recognized at an emotional level is tied to being recognized for “who you are”—in the present study a person defining oneself as LGBTQ+, and as a parent. This can generate a sense of confidence that enables you to act, communicate and take part in close communities (Siisiäinen, 2014). LGBTQ+ persons reported feeling recognized as parents by the nurses through their attitudes and actions, as shown by the present study. At the same time, being regarded as a family also emphasized the understanding of legal recognition. Likewise, Honneth (1995) describes different forms of disrespect, such as moral violence toward fundamental self-confidence, denial of rights and degradation of social life. In this MCHC context, however, to be recognized as parents they had to “come out” as LGBTQ+ (Andersen et al., 2017) and had to justify and explain the family structure and parental roles as shown in several of the included studies (Andersen et al., 2017; Chapman et al., 2012; Dahl & Malterud, 2015; Kerppola et al., 2020; McKelvey, 2014). Similar findings are presented by Bos and Gartrell (2020), where lesbian parents felt they had to defend the quality of their parenting, due to society’s views and attitudes toward sexual orientation. Furthermore, previous negative experiences of discrimination made the LGBTQ+ parents more hesitant, with an approach based on low expectations and a fear of facing judgmental, as the present study indicates. Experiencing prejudiced, heterosexist and judgmental attitudes jeopardizes legal recognition, which influenced their experience of the right to be a family. This made them fearful of not being recognized as parents and gave them a sense of being excluded, which contributed to the LGBTQ+ parents’ questioning and struggle to define a parental self. This also underlines the importance of nursing care for LGBTQ+ parents to experience recognition in their parenthood.

### Participation in Trusting Relations

The synthesis illuminates the importance of participation (Honneth, 1995; Siisiäinen, 2014). LGBTQ+ parents in the present study describe how they experienced a sense of autonomy, of being acknowledged as parents and of having a sense of belonging when they were included in the decision-making, were well-informed, and able to influence their situation. Participation contributed to the development of trusting relations with nurses. Furthermore, participation is linked to health promotion (World Health Organization, 2009) and enabling people to get more control of their lives and health (Rodwell, 1996). Likewise, the experience of belonging to a community was also regarded as an essential element. Our findings show thar connecting with other families with the same or a similar family structure gave the parents an opportunity to exchange experience and show their children that similar families existed. The findings did reveal, however, that some LGBTQ+ parents were reluctant to participate in parental groups, as they did not feel included, due to heteronormative structures and concerns about other parents’ response to them. Leal et al. (2021) highlights the value of assisting with and fostering supportive relationships among LGB parents.

### Power and Language

LGBTQ+ parents face power struggles in the MCHC system, despite the continuous work on ensuring their fundamental rights (FRA - European Union Agency for Fundamental Rights, 2016) and the fact that LGBTQ+ parents’ marriage rights are enacted in national legislation in 30 countries and territories around the world (Masci et al., 2019). The power struggle could be found in different settings, from the relationship between the parents and the nurses, demonstrated by attitudes and actions, to the system’s structure. Halvorsen et al. (2020), describe how empowerment tends to be given by nurses, rather than taken by MCHC users, with limited focus on the power balance within the relationship, which challenges the equality. The present study shows challenges related to language use in written material and how categorization can give a sense of alienation and exclusion, and a feeling of marginalization because the system is too heteronormative. LGBTQ+ parents also expressed how they grapple to identify themselves within the heteronormative categories and structure. This has a negative impact on developing a parental identity, as they found themselves defined by what they were not (non-birth mother) or their own perception of their parental role. This is supported by the findings of Padavic and Butterfield (2011), as they argue that being defined by what you are *not* will weaken the link to motherhood. One must assume that this is similar in other contexts where a parental identity is sought.

In our meta-ethnography, LGBTQ+ parents valued nurses’ efforts to reflect the language used by the family. The impact of language is associated with communication skills. Röndahl et al. (2009) describes how poor communication can affect the quality of care and may lead to feelings of insecurity and the experience of disrespectful treatment. Furthermore, Brennan and Sell (2014) state that nurses can make the transition to parenthood easier by validating and recognizing the maternal role through written and spoken language.

This meta-ethnography highlights how a lack of knowledge about LGBTQ+ families and their needs forced the parents to take on an educator role. LGBTQ+ parents may be perceived as being in a vulnerable position when they seek support and guidance from nurses. Taking this into consideration, one can question whether it is appropriate that LGBTQ+ parents must educate nurses about LGBTQ+ families, or whether this responsibility should be included in the nursing education. Our findings show that knowledge and practical guidance to support LGBTQ+ families’ unique opportunities were highly valued. Previous research has identified the need for increased training of health care professionaøs to improve their ability to provide appropriate services for LGBTQ+ persons and families (Kelsall-Knight, 2021).

Empowerment is derived from interactions, including power sharing, respect and recognizing each other’s strengths (Rodwell, 1996). Our findings highlight how LGBTQ+ parents wished to be recognized for the qualities unique to them as humans, as parents, and included as equals in trusting and participatory relationships in which they can influence their own parenthood.

### Strengths and Limitations

This meta-ethnography has strengths in its rigorous literature search, use of the CASP quality appraisal tool (Critical Appraisal Skills Programme, 2018) and transparency following Noblit and Hare (1988). The research team included both novice and experienced qualitative researchers in a reflective research process to create new knowledge. In the studies included, the sample sizes were small, but represented six different countries on three continents, which facilitated rich data material. However, most of the studies refer to lesbian women coming from educated, middle class and white backgrounds. The participants may be represented by “the pioneers,” while the LGBTQ+ parents who are more vulnerable and less visible by choice will remain unheard. Further research into LGBTQ+ parents’ experiences of parenthood is required, with specific awareness of bisexual, transgender and queer parents. Likewise, gay fathers’ experiences of parenthood should be explored. Lesbian parenthood has gained more research interest, which can make the challenges faced by gay fathers less visible, as the path to parenthood may be more cumbersome for them than for lesbian mothers.

## Conclusion

The lines-of-argument synthesis described by the overarching metaphor “To be recognised as parents, unique and good enough, like everybody else” reflects how LGBTQ+ persons wish to be confirmed and recognized as parents, with unique and universal needs, by nurses and at MCHCs. At the same time, they want to be supported and encouraged for abilities which enable them to embrace parenthood in line with their definition of being a parent. Inclusion and participation are crucial to avoiding a sense of marginalization, judgmental attitudes, and exclusion. Strong power is commanded by language, as words and definitions can strengthen LGBTQ+ parents’ experience of being entitled and regarded as being parents; or on the contrary, can deprive them of the experience of the right to parenthood.

### Implications for Nursing Practice and Education

This meta-ethnography contributes knowledge of LGBTQ+ parents’ experience of parenthood in an MCHC context. The findings can contribute to increased awareness of the impact nurses have in these encounters. The findings help us better understand how nurses can support and accommodate LGBTQ+ parents in being supported and recognized as parents and family.

To provide nursing care to LGBTQ+ parents and families, educational interventions are needed. MCHCs should improve their policies and guidelines to embrace diversity, with greater openness toward variations in gender, parenthood, and family constellations.

## Supplemental Material

sj-docx-1-gqn-10.1177_23333936231181176 – Supplemental material for LGBTQ+ Persons’ Experiences of Parenthood in the Context of Maternal and Child Health Care: A Meta-ethnography Click here for additional data file. Supplemental material, sj-docx-1-gqn-10.1177_23333936231181176 for LGBTQ+ Persons’ Experiences of Parenthood in the Context of Maternal and Child Health Care: A Meta-ethnography by Charlotte Haugland, Bente Kristin Høgmo and Terese Elisabet Bondas in Global Qualitative Nursing Research
