# Supplementary figures and images for: LGBTQ+ Persons’ Experiences of Parenthood in the Context of Maternal and Child Health Care: A Meta-ethnography
Source: Glob Qual Nurs Res. 2023 Jun 20;10:23333936231181176. doi: 10.1177/23333936231181176 (PMC10286167; doi:10.1177/23333936231181176)

## Supplementary file

## CASP - the quality appraisal of the included studies


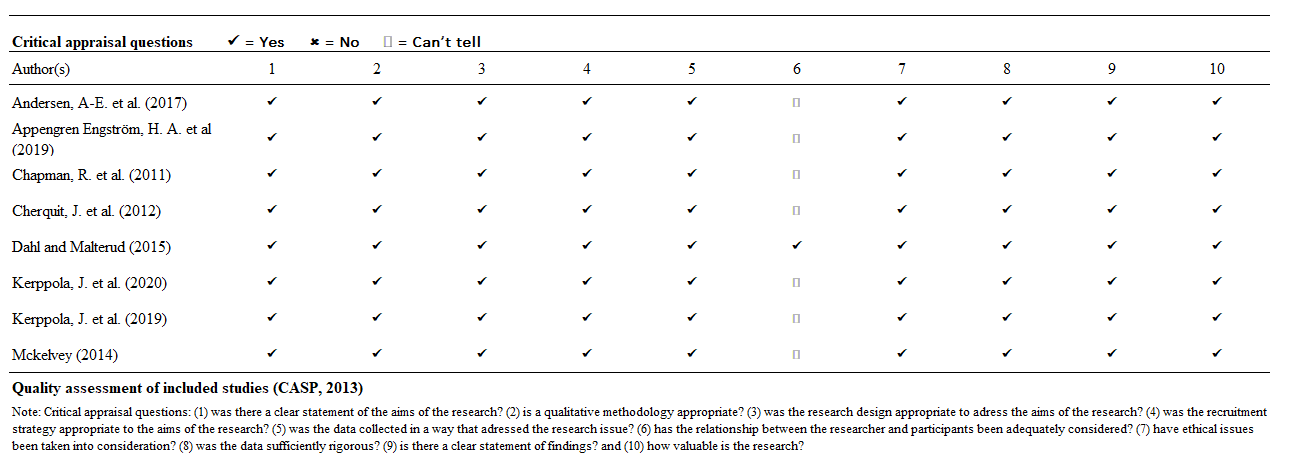


## Table 4 - CASP

Supplement: sj-docx-1-gqn-10.1177_23333936231181176 – Supplemental material for LGBTQ+ Persons’ Experiences of Parenthood in the Context of Maternal and Child Health Care: A Meta-ethnography [file sj-docx-1-gqn-10.1177_23333936231181176.docx]
